# Potential Diagnostic Applications of Multi-Delay Arterial Spin Labeling in Early Alzheimer’s Disease: The Chinese Imaging, Biomarkers, and Lifestyle Study

**DOI:** 10.3389/fnins.2022.934471

**Published:** 2022-07-22

**Authors:** Mengfan Sun, Yan-Li Wang, Runzhi Li, Jiwei Jiang, Yanling Zhang, Wenyi Li, Yuan Zhang, Ziyan Jia, Michael Chappell, Jun Xu

**Affiliations:** ^1^Department of Neurology, Beijing Tiantan Hospital, Capital Medical University, Beijing, China; ^2^China National Clinical Research Center for Neurological Diseases, Beijing Tiantan Hospital, Capital Medical University, Beijing, China; ^3^Department of Radiology, Beijing Tiantan Hospital, Capital Medical University, Beijing, China; ^4^Mental Health and Clinical Neurosciences and Sir Peter Mansfield Imaging Centre, School of Medicine, University of Nottingham, Nottingham, United Kingdom; ^5^Nottingham Biomedical Research Centre, Queen’s Medical Centre, University of Nottingham, Nottingham, United Kingdom

**Keywords:** Alzheimer’s disease, mild cognitive impairment, diagnosis, multi-delay arterial spin labeling, cerebral blood flow, arterial transit time

## Abstract

**Background:**

Cerebral blood flow (CBF) alterations are involved in the onset and progression of Alzheimer’s disease (AD) and can be a potential biomarker. However, CBF measured by single-delay arterial spin labeling (ASL) for discrimination of mild cognitive impairment (MCI, an early stage of AD) was lack of accuracy. Multi-delay ASL can not only provide CBF quantification but also provide arterial transit time (ATT). Unfortunately, the technique was scarcely applied to the diagnosis of AD. Here, we detected the utility of ASL with 1-delay and 7-delay in ten regions of interest (ROIs) to identify MCI and AD.

**Materials and Methods:**

Pseudocontinuous ASL (pCASL) MRI was acquired on a 3T GE scanner in adults from the Chinese Imaging, Biomarkers, and Lifestyle (CIBL) Study of AD cohort, including 26 normal cognition (NC), 37 MCI, and 39 AD. Receiver operating characteristic (ROC) analyses with 1-delay and 7-delay ASL were performed for the identification of MCI and AD. The DeLong test was used to compare ROC curves.

**Results:**

For CBF of 1-delay or 7-delay the AUCs showed moderate-high performance for the AD/NC and AD/MCI comparisons (AUC = 0.83∼0.96) (*p* < 0.001). CBF of 1-delay performed poorly in MCI/NC comparison (AUC = 0.69) (*p* < 0.001), but CBF of 7-delay fared well with an AUC of 0.79 (*p* < 0.001). The combination of CBF and ATT of 7-delay showed higher performance for AD/NC, AD/MCI, and MCI/NC comparisons with AUCs of 0.96, 0.89, and 0.89, respectively (*p* < 0.001). Furthermore, combination of CBF, ATT, sex, age, *APOE* ε4, and education improved further the accuracy (*p* < 0.001). In subgroups analyses, there were no significant differences in CBF of 7-delay ASL for identification of AD or MCI between age subgroups (*p* > 0.05).

**Conclusion:**

The combination of CBF and ATT with 7-delay ASL showed higher performance for identification of MCI than CBF of 1-delay, when adding to sex, age, *APOE* ε4 carrier status, and education years, the diagnostic performance was further increased, presenting a potential imaging biomarker in early AD.

## Introduction

Alzheimer’s disease (AD) is the most common neurodegenerative disorder and is neuropathologically hallmarked by extracellular β-amyloid (Aβ) plaques and by intracellular neurofibrillary tangles consisting of hyperphosphorylated tau protein, which starts 10–20 years before the onset of clinical symptoms (Frisoni et al., 2011; Dubois et al., 2016; Jack et al., 2018). Specific biomarkers of AD, Aβ, and Tau could be detected on positron emission tomography (PET) or in cerebrospinal fluid (CSF), which are expensive, invasive, and limiting widespread application clinically (Verberk et al., 2018). For this reason, studies have increasingly focused on affordable and non-invasive methods for the detection of AD at an earlier stage [such as mild cognitive impairment (MCI) and predementia phase of AD] to delay and prevent the progression of the disease (Alzheimer’s Association, 2018; Thomas et al., 2021). Cerebral blood flow (CBF) changes are part of neurovascular unit impairment that is considered as an essential role in AD pathogenesis (Bell and Zlokovic, 2009; Zlokovic, 2011). The two-hit vascular hypothesis of AD states that cerebrovascular damage (“hit one”), including blood-brain barrier (BBB) breakdown and CBF reductions, contributes to the accumulation of neurotoxic molecules and hypoperfusion that can directly initiate neuronal injury; subsequently, there is Aβ deposition (“hit two”) leading to the onset and progression of AD dementia (Nelson et al., 2016; Kisler et al., 2017). CBF has gained attention, which is measured by arterial spin labeling (ASL) MRI, and ASL is a non-invasive technique using magnetically labeled arterial water as an endogenous tracer (Sierra-Marcos, 2017; Zhang et al., 2017).

Using ASL, several studies have supported the pattern of spread of hypoperfusion in AD starting from the precuneus, spreading to the rest of the parietal cortex and the cingulate gyrus, then the frontal and temporal lobes and eventually the occipital cortex (Dai et al., 2009; Binnewijzend et al., 2013; Wierenga et al., 2014; Xekardaki et al., 2015; Love and Miners, 2016; Duan et al., 2021). In cross-sectional studies, positive correlations have been found between general cognition and cortical CBF in the precuneus and posterior cingulate, parietal, frontal, temporal, and occipital lobes (Binnewijzend et al., 2013; Leeuwis et al., 2017; Duan et al., 2020). Decreased CBF of all of the above-mentioned regions and entorhinal cortex has also been reported to be a useful predictor of future cognitive decline in longitudinal studies (De Vis et al., 2018; Sanchez et al., 2020; Bangen et al., 2021; Duan et al., 2021). Moreover, areas of hypoperfusion measured with ASL have a significant agreement with areas of hypometabolism measured by ^18^F-fluorodeoxyglucose PET (^18^F-FDG PET) in bilateral parietotemporal cortex, precuneus, and posterior cingulate cortex in patients with AD and MCI compared with healthy control participants (Riederer et al., 2018; Dolui et al., 2020); and studies have shown that these imaging modalities had similar associations with amyloid deposition in regions of frontal, parietal, and temporal cortex performed with voxel-wise regression based on cross-sectional analyses (McDade et al., 2014; Yan et al., 2018). Although these studies indicate that ASL-MRI CBF may be a valuable biomarker for AD, only a few studies explored the diagnostic performance for discrimination of MCI stage, which did not show high sensitivity and specificity (Binnewijzend et al., 2013; Xekardaki et al., 2015; Dolui et al., 2020).

Notably, ASL studies in AD to date have mostly been performed using a single post-labeling delay (PLD), the time between labeling and acquisition of the image for evaluating CBF. The main limitation of single-delay ASL is that any variation in arterial transit time (ATT) is ignored. ATT represents the duration for labeled blood to travel from the labeling region to the point of delivery to the brain tissue, which varies widely with vascular pathology and normal aging and can have a significant effect on quantifying CBF (Liu et al., 2012; Wang et al., 2013). Multi-delay ASL, with multiple PLDs, can not only improve the accuracy of CBF quantification by calculating mean values of CBF at each PLD corrected by ATT but also enable the calculation of ATT itself (Yoshiura et al., 2009; Wang et al., 2013). Little is known about how CBF and ATT change with multi-delay ASL in AD and whether the multi-delay is better than single-delay ASL for the identification of MCI.

Considering spiral readout and background suppression, 3D fast-spin-echo pseudocontinuous ASL (pCASL) is recommended as the standard method for ASL image in the clinical setting (Alsop et al., 2015), the current study investigated the changes in CBF and ATT using 7-delay ASL and compared the diagnostic value between 1-delay and 7-delay ASL in ten regions of interest (ROIs): left and right regions of olfactory, posterior cingulate, hippocampus, cuneus, and precuneus. Besides, we also examined associations of cognitive performance with CBF and ATT in ROIs.

## Materials and Methods

### Participants

Subjects (*n* = 102) with ASL-MRI data were included in the Chinese Imaging, Biomarkers, and Lifestyle (CIBL) Study of AD cohort from April to October 2021. They were carefully screened with a medical history, neuropsychological assessment, and brain MRI. In this study, all participants from both genders with minimum primary school education, and were clinically diagnosed with normal cognition (NC), MCI, and AD. Patients with AD fulfilled the clinical criteria of probable AD dementia defined by the National Institute on Aging and Alzheimer’s Association (NIA-AA, 2011) (McKhann et al., 2011). Patients with MCI had memory complaints and fulfilled the criteria defined by NIA-AA (Albert et al., 2011). NC subjects had no cognitive impairment and had Mini-Mental State Examination (MMSE) scores of 25–30 and Montreal Cognitive Assessment (MOCA) scores of 26–30. Subjects with the evidence that might affect cognition, including cerebrovascular diseases (e.g., stroke, multiple infarcts, and severe white matter hyperintensity burden), other neurodegenerative diseases (e.g., frontotemporal dementia and dementia with Lewy bodies), and other neurological diseases (e.g., head injury, hydrocephalus, and encephalitis), were excluded. Subjects who suffered from organ failure, cancer, severe depression, psychiatric illness, drugs or alcohol abuse, and inability to perform MRI scan were also excluded. The study was approved by the Institutional Review Board of Beijing Tiantan Hospital of Capital Medical University (KY2021-028-01). And the CIBL study had been registered at http://www.chictr.org.cn/index.aspx (ChiCTR2100049131), before enrolling participants. Written informed consent was acquired from each participant or their guardians.

### MRI Acquisition

Imaging data were acquired on a 3T MR scanner (SIGNA Premier; GE HealthCare, Milwaukee, WI, United States) with a 48-channel head coil. A high-resolution 3D T1-weighted sequence was acquired with the following parameters: repetition time (TR)/echo time (TE)/inversion time (TI) = 7.3/3.0/450 ms, flip angle = 12^°^, field of view (FOV) = 256 mm × 256 mm, matrix size = 256 × 256, and slice thickness = 1.0 mm. A high-resolution 3D T2-weighted FLAIR sequence was acquired with the following parameters: TR/TE/TI = 5,000/106/1,515 ms, FOV = 256 mm × 256 mm, matrix size = 256 × 256, and slice thickness = 1.0 mm. ASL images were acquired with a background-suppressed 3D stack-of-spirals fast-spin-echo sequence preceded by a Hadamard-encoded pCASL module. ASL imaging parameters with 1-delay were acquired with the following parameters: TR/TE = 4,849/10.6 ms, FOV = 220 mm × 220 mm, slice thickness = 4.0 mm, label duration = 1,450 ms, and PLD = 2,025 ms, with these the proton density image and the perfusion weighted image were generated; and with 7-delay were acquired with the following parameters: pCASL module modified to acquire an additional control-only phase, TR/TE = 7,152/11.2 ms, FOV = 220 mm × 220 mm, slice thickness = 3.0 mm, label durations = 220, 260, 300, 370, 480, 680, and 1,180 ms, and PLDs = 1,000, 1,220, 1,480, 1,780, 2,150, 2,620, and 3,320 ms.

### Arterial Spin Labeling Processing

Arterial spin labeling data were quantitatively analyzed through an automatic software “CereFlow” by AnImage (Beijing) Technology Co., Ltd. with the following steps: (i) brain perfusion images were calculated from the raw ASL data [CBF for 1-delay ASL (Alsop et al., 2015) and CBF/ATT for 7-delay ASL (van der Thiel et al., 2018)]; (ii) the M0 image and the T1-weighted image were coregistered (rigid-body transformation with mutual information as similarity metric optimized with exhaustive method); (iii) T1-weighted image was normalized (non-linear transformation, minimizing the bending energies of the deformation fields, and the residual squared difference) onto Montreal Neurological Institute (MNI) template (Binnewijzend et al., 2013), and the M0 image and the perfusion images were then transformed onto the same space as the template as well; (iv) the gray matter regions were further mapped into different cortical regions by masking with automated anatomical labeling (AAL) atlas (Rolls et al., 2015); and (v) ten ROIs were chosen: left and right of olfactory, posterior cingulate, hippocampus, cuneus, and precuneus from the masked regions.

### Total Gray Matter Volume

Total gray matter volume was acquired through the software “Dr. Brain” by YIWEI medical technology Co., Ltd. (Wei et al., 2020). Briefly, (i) the T1-weighted image was segmented into gray matter, white matter, and CSF; (ii) a template is created using the Dartel algorithm from the subjects’ tissue probability maps obtained at the previous step; (iii) iterate the created template, register the subject’s organization probability map with the previous template in each iteration, average the registered organization probability map again to obtain a new template, and finally register the probability map to MNI space; and (iv) total gray matter was then computed based on neuromorphometrics atlas.

### White Matter Hyperintensity Volume

Detailed methods for WMH volumetric quantification have been previously described (Jiang et al., 2020). First, all T2-weighted FLAIR images uniformly went through preprocessing operations (bias correction and spatial normalization), followed by a segmentation stage where the WMH was delineated. Sequentially, coregistration of T2-weighted FLAIR image with MNI template and Hammers atlas is used to analyze the distribution of WMH.

### Covariates

Demographic information included sex, age, education years, apolipoprotein E (*APOE*) ε4 carrier status, pulse pressure (systolic-diastolic blood pressure), and body mass index (BMI). The presence of *APOE* ε4 genotype was tested by using restriction enzyme isoform genotyping on deoxyribonucleic acid (DNA) extracts. *APOE* ε4 carriers were defined as subjects with at least one ε4 allele (ε4/ε4, ε4/ε3, or ε4/ε2). WMH volume and total gray matter volume were also selected as covariates.

### Statistical Analyses

For continuous variables, differences between groups were analyzed using one-way ANOVA with *post-hoc* least significance difference (LSD) tests. Chi-squared tests were used to compare frequency distributions of categorical variables. Differences in CBF and ATT between diagnostic groups were analyzed using ANOVA with *post-hoc* LSD tests, followed by correction with sex, age, pulse pressure, BMI, WMH volume, and total gray matter volume. Linear regression analyses were performed to assess relationships between CBF or ATT of each ROI (independent variables) and cognition tested by MMSE and MoCA scores (dependent variables) across the diagnostic groups. Sex, age, education years, *APOE* ε4 carrier status, WMH volume, and total gray matter volume were entered into each model as covariates. The area under the curves (AUCs) between 1-delay and 7-delay ASL for identification of AD were compared through receiver operating characteristic (ROC) analyses in leave-one-out cross-validation, in which the predicted values were calculated using a binary logistic regression model. The ROC curves were compared using the DeLong test. All tests were two-tailed, and *p* < 0.05 was considered statistically significant. All statistical analyses were performed using SPSS 24.0 and Medcalc software, and ROC figures were generated with GraphPad Prism 8.0.

## Results

### Participants and Characteristics

Table 1 summarized demographic and clinical data of 102 subjects, namely, 26 NC, 37 MCI, and 39 AD. AD group had significantly older age, lower MMSE and MoCA scores, less BMI and total gray matter volume, and more WMH volume compared with MCI and NC groups (*p* < 0.05). Patients with AD had a higher proportion of *APOE* ε4 carrier status than patients with MCI and had lower education years than NC subjects (*p* < 0.05). MoCA scores and gray matter volume were lower in the MCI group than in the NC group (*p* < 0.05). There were no significant differences in sex, pulse pressure, hypertension, diabetes, and heart disease between groups (*p* > 0.05). Supplementary Figure 1 showed the example images of CBF and ATT changes in NC, MCI, and AD subjects.

**TABLE 1:** Demographic and clinical characteristics of participants.

	NC (*n* = 26)	MCI (*n* = 37)	AD (*n* = 39)	*P*-value^‡^
Age, year	59.77 ± 7.83	63.00 ± 7.06	68.15 ± 8.71*^†^	**<0.001**
Female, *n* (%)	17 (65.4)	28 (75.7)	20 (51.3)	0.085
Education, year	12.35 ± 3.73	11.57 ± 3.36	10.05 ± 3.53*	**0.030**
*APOE* ε4 carrier, *n* (%)	7 (26.9)	4 (10.8)	19^#^ (50.0)^†^	**0.001**
BMI, kg/m^2^	24.40 ± 2.59	24.11 ± 3.90	22.33 ± 3.22*^†^	**0.020**
Pulse pressure, mmHg	43.15 ± 14.17	46.35 ± 10.56	49.59 ± 14.20	0.150
MMSE score	29.04 ± 0.87	27.14 ± 1.57	17.10 ± 6.12*^†^	**<0.001**
MoCA score	27.31 ± 1.49	21.70 ± 1.87*	11.03 ± 5.94*^†^	**<0.001**
Gray matter volume, cm^3^	622.32 ± 61.33	570.56 ± 57.90*	535.37 ± 52.54*^†^	**<0.001**
WMH volume, cm^3^	1.45 ± 2.90	1.62 ± 1.73	5.67 ± 5.47*^†^	**<0.001**
Hypertension, *n* (%)	8 (30.8)	12 (32.4)	10 (25.6)	0.797
Diabetes, *n* (%)	3 (11.5)	8 (21.6)	5 (12.8)	0.457
Heart disease^§^, *n* (%)	2 (7.7)	10 (27.0)	5 (12.8)	0.092

*Data are mean ± standard deviation for continuous variables, and percentage (%) and number (n) of participants for categorical variables. Abbreviations: NC, normal cognition; MCI, mild cognitive impairment; AD, Alzheimer’s disease; APOE, apolipoprotein E; BMI, body mass index; MMSE, mini-mental state examination; MoCA, Montreal cognitive assessment; WMH, white matter hyperintensity; LSD, least significance difference.*

*^‡^Analysis of variance and chi-squared tests.*

*^§^Including coronary atherosclerotic heart disease and heart arrhythmia (such as atrial fibrillation).*

*^#^One patient had missing value.*

Bolded values means the significant differences between AD/MCI/HC comparison. *refers to the significant differences between AD and NC. ^†^refers to the significant differences between AD and MCI.

### Cerebral Blood Flow and Arterial Transit Time Changes

The patients with AD had decreased CBF from 1-delay ASL in all ROIs compared with MCI and NC groups (*p* < 0.05), which consisted of 7-delay ASL except in the left hippocampus (*p* = 0.051) (Table 2). ATT was prolonged in patients with AD compared with MCI and NC groups in each ROI (*p* < 0.05) (Table 3). There were no significant differences between MCI and NC in CBF and ATT (*p* > 0.05). Additional correction for sex, age, WMH volume, total gray matter volume, pulse pressure, and BMI did not change the differences between groups.

**TABLE 2:** Regions of interest (ROI)-based CBF with 1-delay and 7-delay ASL in groups.

	1-delay				7-delay			
	NC	MCI	AD	*P*-value^‡^	NC	MCI	AD	*P*-value^‡^
Olfactory L	46.82 ± 9.41	45.12 ± 10.30	37.19 ± 6.91*^†^	**<0.001**	50.44 ± 10.64	51.74 ± 13.96	43.73 ± 10.70*^†^	**0.011**
Olfactory R	46.62 ± 9.70	44.61 ± 10.13	37.63 ± 6.99*^†^	**<0.001**	49.73 ± 12.10	50.44 ± 14.48	42.55 ± 9.98*^†^	**0.012**
Posterior Cingulate L	67.27 ± 12.77	64.57 ± 18.07	47.06 ± 14.36*^†^	**<0.001**	60.18 ± 13.52	61.00 ± 21.80	43.73 ± 16.22*^†^	**<0.001**
Posterior Cingulate R	56.50 ± 11.12	55.08 ± 14.77	40.67 ± 11.08*^†^	**<0.001**	48.56 ± 12.63	53.63 ± 18.24	38.45 ± 12.42*^†^	**<0.001**
Hippocampus L	43.94 ± 8.79	44.39 ± 9.44	37.13 ± 6.75*^†^	**<0.001**	44.27 ± 9.46	48.69 ± 14.83	38.29 ± 10.22^†^	**0.001**
Hippocampus R	43.23 ± 7.73	42.99 ± 10.29	36.16 ± 7.34*^†^	**0.001**	44.30 ± 9.00	47.83 ± 14.58	37.93 ± 9.53*^†^	**0.001**
Cuneus L	52.86 ± 10.66	51.90 ± 16.79	37.06 ± 12.11*^†^	**<0.001**	53.94 ± 13.87	55.66 ± 20.59	38.65 ± 12.23*^†^	**<0.001**
Cuneus R	53.22 ± 12.15	52.91 ± 17.29	38.33 ± 13.69*^†^	**<0.001**	52.18 ± 13.87	56.58 ± 21.71	39.62 ± 13.85*^†^	**<0.001**
Precuneus L	54.40 ± 10.48	53.02 ± 15.07	40.33 ± 10.60*^†^	**<0.001**	55.45 ± 13.73	56.10 ± 19.70	40.47 ± 13.01*^†^	**<0.001**
Precuneus R	53.57 ± 9.62	52.32 ± 14.07	38.88 ± 10.54*^†^	**<0.001**	52.42 ± 12.42	55.00 ± 18.07	39.53 ± 11.18*^†^	**<0.001**

*Data are mean ± standard deviation (in milliliters per 100 g/min). Abbreviations: NC, normal cognition; MCI, mild cognitive impairment; AD, Alzheimer’s disease; ROI, region of interest; CBF, cerebral blood flow; ASL, arterial spin labeling; L, left; R, right; LSD, least significance difference.*

*^‡^Analysis of variance.*

*p < 0.05 compared to the NC group with LSD tests.

*^†^p < 0.05 compared to the MCI group with LSD tests.*

**TABLE 3:** Region of interest (ROI)-based ATT with 7-delay ASL in groups.

	NC	MCI	AD	*P*-value^‡^
Olfactory L	1.28 ± 0.16	1.29 ± 0.13	1.38 ± 0.25*^†^	**0.037**
Olfactory R	1.30 ± 0.19	1.27 ± 0.11	1.42 ± 0.24*^†^	**0.003**
Posterior Cingulate L	1.60 ± 0.19	1.57 ± 0.20	1.71 ± 0.19*^†^	**0.005**
Posterior Cingulate R	1.52 ± 0.17	1.52 ± 0.19	1.64 ± 0.20*^†^	**0.009**
Hippocampus L	1.34 ± 0.14	1.34 ± 0.14	1.49 ± 0.20*^†^	**<0.001**
Hippocampus R	1.40 ± 0.16	1.37 ± 0.14	1.49 ± 0.20*^†^	**0.008**
Cuneus L	1.73 ± 0.20	1.71 ± 0.20	1.86 ± 0.20*^†^	**0.003**
Cuneus R	1.77 ± 0.18	1.72 ± 0.20	1.87 ± 0.20*^†^	**0.003**
Precuneus L	1.70 ± 0.18	1.65 ± 0.18	1.80 ± 0.19*^†^	**0.002**
Precuneus R	1.66 ± 0.18	1.64 ± 0.18	1.78 ± 0.20*^†^	**0.003**

*Data are mean ± standard deviation (in seconds). Abbrevaitions: NC, normal cognition; MCI, mild cognitive impairment; AD, Alzheimer’s disease; ROI, region of interest; ATT, arterial transit time; ASL, arterial spin labeling; L, left; R, right; LSD, least significance difference.*

*^‡^Analysis of variance.*

*p < 0.05 compared to the NC group with LSD tests.

*^†^p < 0.05 compared to the MCI group with LSD tests.*

### Associations of Cognitive Performance With Cerebral Blood Flow and Arterial Transit Time

Across diagnostic groups, CBFs of posterior cingulate, cuneus, and precuneus in 1-delay ASL were associated with MMSE and MoCA adjusted for sex, age, education years, *APOE* ε4 carrier status, gray matter volume, and WMH volume (β = 0.207∼0.306, *p* < 0.05) (Supplementary Table 1). This relationship in 7-delay had appeared in the left posterior cingulate, left hippocampus, cuneus, and precuneus (β = 0.186∼0.305, *p* < 0.05 except for the relationship between CBF in cuneus and MMSE) (Supplementary Table 1). Regional ATT of right olfactory, left hippocampus, and right hippocampus was significantly correlated with MMSE (β = −0.212, −0.193, and −0.204, respectively, *p* < 0.05). There was no significant relationship between regional ATT and MoCA (*p* > 0.05).

### Receiver Operating Characteristic Analyses

**FIGURE 1:**
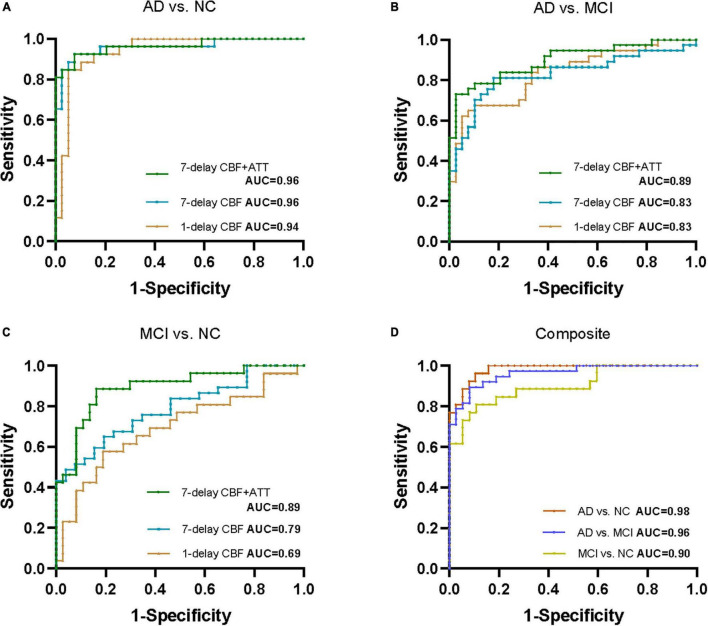
Receiver operating characteristic (ROC) analyses for perfusion parameters of 1-delay and 7-delay ASL in all ROIs in distinguishing different stages of AD. ROC curves for CBF of 1-delay, CBF of 7-delay, or the combination of CBF and ATT of 7-delay in differentiating AD from NC **(A)** and MCI **(B)** and MCI from NC **(C)**. **(D)** AUCs for the combination of CBF and ATT of 7-delay, sex, age, *APOE* ε4 carrier status, and education years (composite) in NC, patients with AD and MCI. NC, normal cognition; MCI, mild cognitive impairment; AD, Alzheimer’s disease; *APOE*, apolipoprotein E; ROI, region of interest; CBF, cerebral blood flow; ATT, arterial transit time; AUC, area under the curve; ROC, receiver operating characteristic.

To compare 1-delay and 7-delay ASL for the diagnostic accuracy, we applied ROC analyses on perfusion parameters in all ROIs. As a result, for CBF of 1-delay the AUCs for the AD/NC and AD/MCI comparisons were 0.94 (*p* < 0.001) and 0.83 (*p* < 0.001), respectively, which were similar to CBF of 7-delay (AUC = 0.96, *p* < 0.001 for AD/NC comparison; AUC = 0.83, *p* < 0.001 for AD/MCI comparison) (Figures 1A,B and Supplementary Table 2). While the AUC for CBF of 7-delay in MCI/NC comparison was higher (AUC = 0.79, *p* < 0.001) compared with that for CBF of 1-delay (AUC = 0.69, *p* < 0.001) (Figure 1C and Supplementary Table 2). We compared the diagnostic capacities of combination of CBF and ATT of 7-delay and found that was more discriminative performance for AD/NC, AD/MCI, and MCI/NC comparisons with AUCs of 0.96 (*p* < 0.001), 0.89 (*p* < 0.001), and 0.89 (*p* < 0.001), respectively (Figures 1A–C and Supplementary Table 2). Furthermore, the AUCs of combination of CBF and ATT of 7-delay, sex, age, *APOE* ε4 carrier status, and educational years (as a composite biomarker) were higher than those mentioned above (AUC = 0.98, *p* < 0.001 for AD/NC comparison; AUC = 0.96, *p* < 0.001 for AD/MCI comparison; and AUC = 0.90, *p* < 0.001 for MCI/NC comparison) (Figure 1D and Supplementary Table 2). In comparison of AUCs between groups, there were no differences in AUCs between AD and NC groups (*p* > 0.05). The AUC of composite showed higher diagnostic efficiency than other methods in AD/MCI comparison (*p* < 0.05). The AUCs of CBF and ATT of 7-delay and composite were more powerful than that of CBF in 1-delay for MCI/NC identification (*p* < 0.05) (Supplementary Table 3).

### Subgroup Receiver Operating Characteristic Analyses by Age

We performed subgroup ROC analyses according to age (middle-aged group [<65 years] and old-aged group [≥65 years]) in CBF of 7-delay ASL. CBF of 7-delay ASL in middle-age group had a significantly discriminative performance for the AD/NC, AD/MCI, and MCI/NC comparisons (AUC = 0.97, 0.95, and 0.83, respectively, *p* < 0.05). The method still had significantly diagnostic performance in old-age group (AUC = 0.97, 0.78, and 0.94, respectively, *p* < 0.05). Besides, there were no significant differences in CBF of 7-delay ASL for identification of AD or MCI between age subgroups (*p* > 0.05) (Supplementary Table 4).

## Discussion

Our study revealed significant CBF decrease and ATT prolongation in the ten ROIs in patients with AD. Correlation analysis showed a strong association between regional CBF and cognitive function across the diagnostic groups. Combining CBF and ATT of 7-delay ASL based on ROIs showed a higher performance for the differentiation of MCI compared with CBF of 1-delay.

The regional hypoperfusion changes measured by 1-delay ASL of patients with AD compared with patients with MCI and NC subjects in this study were consistent with previous studies (Asllani et al., 2008; Dai et al., 2009; Binnewijzend et al., 2013; Ma et al., 2017), similar to CBF changes measured with 7-delay ASL, supporting the theory that patients with AD have a greater decrease of CBF in the widespread brain than MCI and NC groups. It was found that patients with MCI exhibited decreased CBF in the precuneus and posterior cingulate compared to healthy control or subjective cognitive impairment subjects (Dai et al., 2009; Binnewijzend et al., 2013; Soman et al., 2021). Dai et al. (2009) observed increased CBF in the hippocampus and limbic system in patients with MCI, which suggests a compensatory mechanism during the MCI stage of AD. However, our results showed no significant differences in CBF of these ROIs measured by 1-delay or 7-delay ASL in patients with MCI compared to NC. Another prominent abnormality using 7-delay ASL was a significant prolongation of ATT in patients with AD compared to MCI and NC subjects. Such findings might support the hypothesis that there is a neurovascular impairment with the AD pathologic process (Østergaard et al., 2013; Love and Miners, 2016; Nelson et al., 2016). While Yoshiura et al. (2009) using multi-TI pulsed ASL reported patients with AD had no significant ATT prolongation in the hypoperfusion area of the bilateral precunei and the left posterior cingulate compared to NC. The lower signal-to-noise ratio with pulsed ASL and different sample sizes might contribute to the discrepancy in our results (Alsop et al., 2015).

There were positive correlations between CBF with 1-delay and MMSE or MoCA in the posterior cingulate, precuneus, and cuneus with all subjects after adjusting for sex, age, education years, *APOE* ε4 carrier status, total gray matter volume, and WMH volume, consistent with the previous studies (Binnewijzend et al., 2013; Liu et al., 2015; Soman et al., 2021; Zhang et al., 2021). We also found the association between CBF with 7-delay and cognition was prominent in the left posterior cingulate, left hippocampus, left cuneus, and precuneus; and ATT was negatively associated with MMSE but not MoCA in the hippocampus and right olfactoffff0100a0210000ffff0100a0210000ry. The results may suggest that multi-delay ASL can also add value to objective cognitive evaluation.

According to previous literature, CBF in the precuneus and posterior cingulate using pCASL identified patients with AD from NC or subjective cognitive impairment with an AUC up to 0.80, but the predicted probability was poor in distinguishing MCI from AD or NC with AUCs of 0.59 and 0.78, respectively (Binnewijzend et al., 2013; Thomas et al., 2019). Liu et al. (2015) found similar AUC for CBF of posterior cingulate measured by pCASL with PLD of 1.5 s and 2.5 s in AD and NC (0.891 and 0.882, respectively), whereas Dolui et al. (2020) reported AUC of 0.77 for CBF in the same region using pCASL with PLD of 1.5 s. In the present study, given the pattern of spread of hypoperfusion in AD starting from the precuneus, spreading to the rest of the parietal cortex and the cingulate gyrus, then the frontal and temporal lobes, we selected the ten ROIs, namely, the left and right regions of olfactory, posterior cingulate, hippocampus, cuneus, and precuneus to perform ROC analyses (Dai et al., 2009; Binnewijzend et al., 2013; Wierenga et al., 2014; Xekardaki et al., 2015; Love and Miners, 2016; Duan et al., 2021). As a result, ROIs-based CBF with 1-delay performed well in distinguishing AD from MCI and NC, but not MCI from NC. A similar observation was found in predicting AD with 7-delay pCASL, with higher performance in differentiating MCI from NC with an AUC of 0.79. Combining CBF and ATT with 7-delay showed higher discriminatory power in predicting AD or MCI with AUCs up to 90%, when adding to sex, age, *APOE* ε4 carrier status, and education years, the diagnostic performance was further increased. Our study indicated that the combination of regional CBF and ATT of the ten ROIs measured by 7-delay pCASL could be a potential sensitive biomarker for the identification of early AD.

Our study had several limitations. First, the sample size was limited to a single center and some analyses may lack sufficient power. More subjects need to be recruited from multi-centers to validate the current findings. Second, the diagnosis of MCI or AD was based on clinical symptoms and neuropsychological assessment with no evidence of specific biomarkers for Aβ and tau pathology. However, we screened subjects carefully with a medical history, detailed neuropsychological evaluations to exclude those with MCI or dementia not due to AD, and the final consensus diagnosis was decided by the experienced neurologists. Of course, further studies will perform on subjects with pathological biomarkers of AD. Finally, partial volume correction (PVC) was not performed for the measurement of CBF, which confounds the evaluation of perfusion due to brain atrophy (Zhang et al., 2017; Chappell et al., 2021). Our results have shown the high differentiation performance for patients with AD, possibly owing to the additive discriminatory effect of cortical atrophy (Østergaard et al., 2013; Wierenga et al., 2014; Kisler et al., 2017; Ma et al., 2017), indicating that PVC may not necessary for the identification of AD. However, further research is warranted to use PVC with ASL to explore the CBF changes and diagnostic performance for NC subjects and patients with MCI because of the complex changes in atrophy and in perfusion with age and disease (Chen et al., 2011).

## Conclusion

Our study showed patients with AD had apparent CBF decrease and ATT prolongation in ROIs using multi-delay ASL, supporting that there is a neurovascular impairment with the AD pathologic process. The combination of CBF and ATT with 7-delay ASL in the ten ROIs showed a higher diagnostic performance for MCI than CBF of 1-delay, when adding to sex, age, *APOE* ε4 carrier status, and education years, the diagnostic performance was further increased, presenting a potential imaging biomarker in early AD.

## Data Availability Statement

The raw data supporting the conclusions of this article will be made available by the authors, without undue reservation.

## Ethics Statement

The studies involving human participants were reviewed and approved by the Research Ethics Committee of Beijing Tiantan Hospital, Capital Medical University. The patients/participants provided their written informed consent to participate in this study. Written informed consent was obtained from the individual(s) for the publication of any potentially identifiable images or data included in this article.

## Author Contributions

MS wrote the original draft. Y-LW performed the statistical analyses and reviewed the manuscript. RL, JJ, and YaZ designed the study and reviewed the manuscript. WL, YuZ, and ZJ organized the database. MC and JX reviewed and finalized the manuscript. All authors contributed to manuscript revision, read, and approved the submitted version.

## Conflict of Interest

The authors declare that the research was conducted in the absence of any commercial or financial relationships that could be construed as a potential conflict of interest.

## Publisher’s Note

All claims expressed in this article are solely those of the authors and do not necessarily represent those of their affiliated organizations, or those of the publisher, the editors and the reviewers. Any product that may be evaluated in this article, or claim that may be made by its manufacturer, is not guaranteed or endorsed by the publisher.

## References

[B1] Albert M. S. DeKosky S. T. Dickson D. Dubois B. Feldman H. H. Fox N. C. (2011). The diagnosis of mild cognitive impairment due to Alzheimer’s disease: recommendations from the National Institute on Aging-Alzheimer’s Association workgroups on diagnostic guidelines for Alzheimer’s disease. *Alzheimers Dement.* 7 270–279. 10.1016/j.jalz.2011.03.008 21514249 PMC3312027

[B2] Alsop D. C. Detre J. A. Golay X. Günther M. Hendrikse J. Hernandez-Garcia L. (2015). Recommended implementation of arterial spin-labeled perfusion MRI for clinical applications: a consensus of the ISMRM perfusion study group and the European consortium for ASL in dementia. *Magn. Reson. Med.* 73 102–116. 10.1002/mrm.25197 24715426 PMC4190138

[B3] Alzheimer’s Association (2018). 2018 Alzheimer’s disease facts and figures. *Alzheimers Dement.* 14 367–429. 10.1016/j.jalz.2018.02.001

[B4] Asllani I. Habeck C. Scarmeas N. Borogovac A. Brown T. R. Stern Y. (2008). Multivariate and univariate analysis of continuous arterial spin labeling perfusion MRI in Alzheimer’s disease. *J. Cereb. Blood Flow Metab.* 28 725–736. 10.1038/sj.jcbfm.9600570 17960142 PMC2711077

[B5] Bangen K. J. Thomas K. R. Sanchez D. L. Edmonds E. C. Weigand A. J. Delano-Wood L. (2021). Entorhinal perfusion predicts future memory decline, neurodegeneration, and white matter hyperintensity progression in older adults. *J. Alzheimers Dis.* 81 1711–1725. 10.3233/JAD-201474 33967041 PMC9462657

[B6] Bell R. D. Zlokovic B. V. (2009). Neurovascular mechanisms and blood-brain barrier disorder in Alzheimer’s disease. *Acta Neuropathol.* 118 103–113. 10.1007/s00401-009-0522-3 19319544 PMC2853006

[B7] Binnewijzend M. A. A. Kuijer J. P. A. Benedictus M. R. van der Flier W. M. Wink A. M. Wattjes M. P. (2013). Cerebral blood flow measured with 3D pseudocontinuous arterial spin-labeling MR imaging in Alzheimer disease and mild cognitive impairment: a marker for disease severity. *Radiology* 267 221–230. 10.1148/radiol.12120928 23238159

[B8] Chappell M. A. McConnell F. A. K. Golay X. Günther M. Hernandez-Tamames J. A. van Osch M. J. (2021). Partial volume correction in arterial spin labeling perfusion MRI: a method to disentangle anatomy from physiology or an analysis step too far? *Neuroimage* 238:118236. 10.1016/j.neuroimage.2021.118236 34091034

[B9] Chen J. J. Rosas H. D. Salat D. H. (2011). Age-associated reductions in cerebral blood flow are independent from regional atrophy. *Neuroimage* 55 468–478. 10.1016/j.neuroimage.2010.12.032 21167947 PMC3435846

[B10] Dai W. Lopez O. L. Carmichael O. T. Becker J. T. Kuller L. H. Gach H. M. (2009). Mild cognitive impairment and alzheimer disease: patterns of altered cerebral blood flow at MR imaging. *Radiology* 250 856–866. 10.1148/radiol.2503080751 19164119 PMC2680168

[B11] De Vis J. B. Peng S. L. Chen X. Li Y. Liu P. Sur S. (2018). Arterial-spin-labeling (ASL) perfusion MRI predicts cognitive function in elderly individuals: a 4-year longitudinal study. *J. Magn. Reson. Imaging* 48 449–458. 10.1002/jmri.25938 29292540 PMC6028323

[B12] Dolui S. Li Z. Nasrallah I. M. Detre J. A. Wolk D. A. (2020). Arterial spin labeling versus 18F-FDG-PET to identify mild cognitive impairment. *Neuroimage Clin.* 25:102146. 10.1016/j.nicl.2019.102146 31931403 PMC6957781

[B13] Duan W. Sehrawat P. Balachandrasekaran A. Bhumkar A. B. Boraste P. B. Becker J. T. (2020). Cerebral blood flow is associated with diagnostic class and cognitive decline in Alzheimer’s disease. *J. Alzheimers Dis.* 76 1103–1120. 10.3233/JAD-200034 32597803 PMC7970411

[B14] Duan W. Zhou G. D. Balachandrasekaran A. Bhumkar A. B. Boraste P. B. Becker J. T. (2021). Cerebral blood flow predicts conversion of mild cognitive impairment into Alzheimer’s disease and cognitive decline: an arterial spin labeling follow-up study. *J. Alzheimers Dis.* 82 293–305. 10.3233/JAD-210199 34024834 PMC8527573

[B15] Dubois B. Hampel H. Feldman H. H. Scheltens P. Aisen P. Andrieu S. (2016). Preclinical Alzheimer’s disease: definition, natural history, and diagnostic criteria. *Alzheimers Dement.* 12 292–323. 10.1016/j.jalz.2016.02.002 27012484 PMC6417794

[B16] Frisoni G. B. Winblad B. O’Brien J. T. (2011). Revised NIA-AA criteria for the diagnosis of Alzheimer’s disease: a step forward but not yet ready for widespread clinical use. *Int. Psychogeriatr.* 23 1191–1196. 10.1017/S1041610211001220 21813035

[B17] Jack C. R. Bennett D. A. Blennow K. Carrillo M. C. Dunn B. Haeberlein S. B. (2018). NIA-AA research framework: toward a biological definition of Alzheimer’s disease. *Alzheimers Dement.* 14 535–562. 10.1016/j.jalz.2018.02.018 29653606 PMC5958625

[B18] Jiang W. Lin F. Zhang J. Zhan T. Cao P. Wang S. (2020). Deep-learning-based segmentation and localization of white matter hyperintensities on magnetic resonance images. *Interdiscip. Sci.* 12 438–446. 10.1007/s12539-020-00398-0 33140170

[B19] Kisler K. Nelson A. R. Montagne A. Zlokovic B. V. (2017). Cerebral blood flow regulation and neurovascular dysfunction in Alzheimer disease. *Nat. Rev. Neurosci.* 18 419–434. 10.1038/nrn.2017.48 28515434 PMC5759779

[B20] Leeuwis A. E. Benedictus M. R. Kuijer J. P. A. Binnewijzend M. A. A. Hooghiemstra A. M. Verfaillie S. C. J. (2017). Lower cerebral blood flow is associated with impairment in multiple cognitive domains in Alzheimer’s disease. *Alzheimers Dement.* 13 531–540. 10.1016/j.jalz.2016.08.013 27693109

[B21] Liu Y. Zeng X. Wang Z. Zhang N. Fan D. Yuan H. (2015). Different post label delay cerebral blood flow measurements in patients with Alzheimer’s disease using 3D arterial spin labeling. *Magn. Reson. Imaging* 33 1019–1025. 10.1016/j.mri.2015.05.001 26113261

[B22] Liu Y. Zhu X. Feinberg D. Guenther M. Gregori J. Weiner M. W. (2012). Arterial spin labeling MRI study of age and gender effects on brain perfusion hemodynamics. *Magn. Reson. Med.* 68 912–922. 10.1002/mrm.23286 22139957

[B23] Love S. Miners J. S. (2016). Cerebrovascular disease in ageing and Alzheimer’s disease. *Acta Neuropathol.* 131 645–658. 10.1007/s00401-015-1522-0 26711459 PMC4835514

[B24] Ma H. R. Pan P. L. Sheng L. Q. Dai Z. Y. Di Wang G. Luo R. (2017). Aberrant pattern of regional cerebral blood flow in Alzheimer’s disease: a voxel-wise meta-analysis of arterial spin labeling MR imaging studies. *Oncotarget* 8 93196–93208. 10.18632/oncotarget.21475 29190989 PMC5696255

[B25] McDade E. Kim A. James J. Sheu L. K. Kuan D. C.-H. Minhas D. (2014). Cerebral perfusion alterations and cerebral amyloid in autosomal dominant Alzheimer disease. *Neurology* 83 710–717. 10.1212/WNL.0000000000000721 25031286 PMC4150128

[B26] McKhann G. M. Knopman D. S. Chertkow H. Hyman B. T. Jack C. R. Kawas C. H. (2011). The diagnosis of dementia due to Alzheimer’s disease: recommendations from the National Institute on Aging-Alzheimer’s Association workgroups on diagnostic guidelines for Alzheimer’s disease. *Alzheimers Dement.* 7 263–269. 10.1016/j.jalz.2011.03.005 21514250 PMC3312024

[B27] Nelson A. R. Sweeney M. D. Sagare A. P. Zlokovic B. V. (2016). Neurovascular dysfunction and neurodegeneration in dementia and Alzheimer’s disease. *Biochim. Biophys. Acta* 1862 887–900. 10.1016/j.bbadis.2015.12.016 26705676 PMC4821735

[B28] Østergaard L. Aamand R. Gutiérrez-Jiménez E. Ho Y.-C. L. Blicher J. U. Madsen S. M. (2013). The capillary dysfunction hypothesis of Alzheimer’s disease. *Neurobiol. Aging* 34 1018–1031. 10.1016/j.neurobiolaging.2012.09.011 23084084

[B29] Riederer I. Bohn K. P. Preibisch C. Wiedemann E. Zimmer C. Alexopoulos P. (2018). Alzheimer disease and mild cognitive impairment: integrated pulsed arterial spin-labeling MRI and 18F-FDG PET. *Radiology* 288 198–206. 10.1148/radiol.2018170575 29762090

[B30] Rolls E. T. Joliot M. Tzourio-Mazoyer N. (2015). Implementation of a new parcellation of the orbitofrontal cortex in the automated anatomical labeling atlas. *Neuroimage* 122 1–5. 10.1016/j.neuroimage.2015.07.075 26241684

[B31] Sanchez D. L. Thomas K. R. Edmonds E. C. Bondi M. W. Bangen K. J. (2020). Regional hypoperfusion predicts decline in everyday functioning at three-year follow-up in older adults without dementia. *J. Alzheimers Dis.* 77 1291–1304. 10.3233/JAD-200490 32831202 PMC9491307

[B32] Sierra-Marcos A. (2017). Regional cerebral blood flow in mild cognitive impairment and Alzheimer’s disease measured with arterial spin labeling magnetic resonance imaging. *Int. J. Alzheimers Dis.* 2017:5479597. 10.1155/2017/5479597 28573062 PMC5442339

[B33] Soman S. Raghavan S. Rajesh P. G. Varma R. P. Mohanan N. Ramachandran S. S. (2021). Relationship between cerebral perfusion on arterial spin labeling (ASL) MRI with brain volumetry and cognitive performance in mild cognitive impairment and dementia due to alzheimer’s disease. *Ann. Indian Acad. Neurol.* 24 559–565. 10.4103/aian.AIAN_848_20 34728951 PMC8513975

[B34] Thomas B. Sheelakumari R. Kannath S. Sarma S. Menon R. N. (2019). Regional cerebral blood flow in the posterior cingulate and precuneus and the entorhinal cortical atrophy score differentiate mild cognitive impairment and dementia due to Alzheimer disease. *AJNR Am. J. Neuroradiol.* 40 1658–1664. 10.3174/ajnr.A6219 31515217 PMC7028557

[B35] Thomas K. R. Osuna J. R. Weigand A. J. Edmonds E. C. Clark A. L. Holmqvist S. (2021). Regional hyperperfusion in older adults with objectively-defined subtle cognitive decline. *J. Cereb. Blood Flow Metab.* 41 1001–1012. 10.1177/0271678X20935171 32615887 PMC8054731

[B36] van der Thiel M. Rodriguez C. Giannakopoulos P. Burke M. X. Lebel R. M. Gninenko N. (2018). Brain perfusion measurements using multidelay arterial spin-labeling are systematically biased by the number of delays. *AJNR Am. J. Neuroradiol.* 39 1432–1438. 10.3174/ajnr.A5717 29976831 PMC7410552

[B37] Verberk I. M. W. Slot R. E. Verfaillie S. C. J. Heijst H. Prins N. D. van Berckel B. N. M. (2018). Plasma amyloid as prescreener for the earliest alzheimer pathological changes. *Ann. Neurol.* 84 648–658. 10.1002/ana.25334 30196548 PMC6282982

[B38] Wang D. J. J. Alger J. R. Qiao J. X. Gunther M. Pope W. B. Saver J. L. (2013). Multi-delay multi-parametric arterial spin-labeled perfusion MRI in acute ischemic stroke – comparison with dynamic susceptibility contrast enhanced perfusion imaging. *Neuroimage Clin.* 3 1–7. 10.1016/j.nicl.2013.06.017 24159561 PMC3791289

[B39] Wei Y. Huang N. Liu Y. Zhang X. Wang S. Tang X. (2020). Hippocampal and amygdalar morphological abnormalities in Alzheimer’s disease based on three chinese MRI datasets. *Curr. Alzheimer Res.* 17 1221–1231. 10.2174/1567205018666210218150223 33602087

[B40] Wierenga C. E. Hays C. C. Zlatar Z. Z. (2014). Cerebral blood flow measured by arterial spin labeling MRI as a preclinical marker of Alzheimer’s disease. *J. Alzheimers Dis.* 42 (Suppl. 4), S411–S419. 10.3233/JAD-141467 25159672 PMC5279221

[B41] Xekardaki A. Rodriguez C. Montandon M.-L. Toma S. Tombeur E. Herrmann F. R. (2015). Arterial spin labeling may contribute to the prediction of cognitive deterioration in healthy elderly individuals. *Radiology* 274 490–499. 10.1148/radiol.14140680 25291458

[B42] Yan L. Liu C. Y. Wong K.-P. Huang S.-C. Mack W. J. Jann K. (2018). Regional association of pCASL-MRI with FDG-PET and PiB-PET in people at risk for autosomal dominant Alzheimer’s disease. *Neuroimage Clin.* 17 751–760. 10.1016/j.nicl.2017.12.003 29527482 PMC5842754

[B43] Yoshiura T. Hiwatashi A. Yamashita K. Ohyagi Y. Monji A. Takayama Y. (2009). Simultaneous measurement of arterial transit time, arterial blood volume, and cerebral blood flow using arterial spin-labeling in patients with Alzheimer disease. *AJNR Am. J. Neuroradiol.* 30 1388–1393. 10.3174/ajnr.A1562 19342545 PMC7051557

[B44] Zhang N. Gordon M. L. Goldberg T. E. (2017). Cerebral blood flow measured by arterial spin labeling MRI at resting state in normal aging and Alzheimer’s disease. *Neurosci. Biobehav. Rev.* 72 168–175. 10.1016/j.neubiorev.2016.11.023 27908711

[B45] Zhang Q. Wang Q. He C. Fan D. Zhu Y. Zang F. (2021). Altered regional cerebral blood flow and brain function across the Alzheimer’s disease spectrum: a potential biomarker. *Front. Aging Neurosci.* 13:630382. 10.3389/fnagi.2021.630382 33692680 PMC7937726

[B46] Zlokovic B. V. (2011). Neurovascular pathways to neurodegeneration in Alzheimer’s disease and other disorders. *Nat. Rev. Neurosci.* 12 723–738. 10.1038/nrn3114 22048062 PMC4036520

