# Supplementary material for: Potential Diagnostic Applications of Multi-Delay Arterial Spin Labeling in Early Alzheimer’s Disease: The Chinese Imaging, Biomarkers, and Lifestyle Study
Source: Front Neurosci. 2022 Jul 22;16:934471. doi: 10.3389/fnins.2022.934471 (PMC9353523; doi:10.3389/fnins.2022.934471)
Supplement: Supplementary file 1 [file Data_Sheet_1.docx]

**Supplementary Material**

**
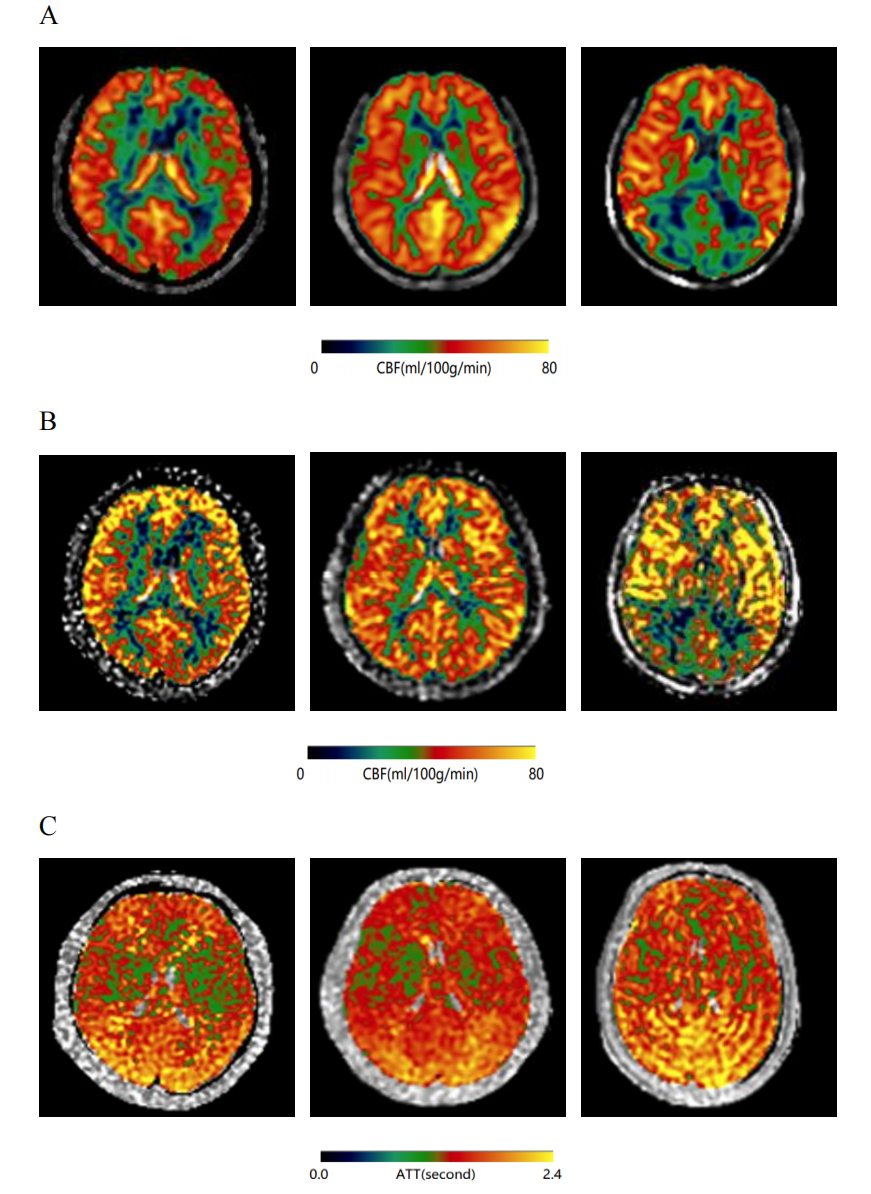
**

**Fig. S1** Maps of CBF of 1-delay (A), CBF of 7-delay (B), and ATT of 7-delay (C) ASL in a 55-year-old woman of NC (left), a 58-year-old woman of MCI (middle), and a 77-year-old woman of AD (right). Note the AD patient had CBF decrease and ATT prolongation in the bilateral posterior border zones compared to MCI and NC subjects. Abbreviations: NC, normal cognition; MCI, mild cognitive impairment; AD, Alzheimer’s disease; CBF, cerebral blood flow; ATT, arterial transit time.

**Table S1** Correlation of based-ROI CBF with MMSE and MoCA

|  | MMSE | |  | | MoCA | |
| --- | --- | --- | --- | --- | --- | --- |
|  | 1-delay | 7-delay | | 1-delay | | 7-delay |
| Olfactory L | 0.165 | 0.053 | | 0.155 | | 0.05 |
| Olfactory R | 0.144 | 0.021 | | 0.171 | | 0.053 |
| Posterior Cingulate L | 0.271^†^ | 0.235^*^ | | 0.251^†^ | | 0.237^†^ |
| Posterior Cingulate R | 0.230^*^ | 0.172 | | 0.218^*^ | | 0.151 |
| Hippocampus L | 0.141 | 0.219 ^*^ | | 0.137 | | 0.195^*^ |
| Hippocampus R | 0.140 | 0.116 | | 0.152 | | 0.129 |
| Cuneus L | 0.306^†^ | 0.305^†^ | | 0.304^†^ | | 0.309^†^ |
| Cuneus R | 0.210^*^ | 0.192 | | 0.207^*^ | | 0.186^*^ |
| Precuneus L | 0.295^†^ | 0.258^*^ | | 0.298^†^ | | 0.267^†^ |
| Precuneus R | 0.236^*^ | 0.207^*^ | | 0.245^*^ | | 0.213^*^ |

Data are standardized β values and were adjusted for sex, age, education years, *APOE ε*4 carrier status, gray matter volume and WMH volume. Abbreviations: NC, normal cognition; MCI, mild cognitive impairment; AD, Alzheimer’s disease; MMSE, Mini-Mental State Examination; MoCA, Montreal Cognitive Assessment; ROI, region of interest; CBF, cerebral blood flow; L, left; R, right.

^*^ *p* <0.05.

^†^*p* <0.01.

**Table S2** ROC analyses from logistic regression models for identifying different stages of AD in different models

|  |  | AUC (95% CI) | *p-*value | Sensitivity | Specificity |
| --- | --- | --- | --- | --- | --- |
| AD vs. NC | Model 1 | 0.96(0.91, 1.00) | **< 0.001** | 0.92 | 0.92 |
|  | Model 2 | 0.96 (0.91, 1.00) | **< 0.001** | 0.92 | 0.92 |
|  | Model 3 | 0.94(0.88, 0.99) | **< 0.001** | 0.85 | 0.95 |
|  | Model 4 | 0.98 (0.96, 1.00) | **< 0.001** | 0.96 | 0.90 |
| AD vs. MCI | Model 1 | 0.89 (0.83, 0.97) | **< 0.001** | 0.73 | 0.97 |
|  | Model 2 | 0.83 (0.73, 0.93) | **< 0.001** | 0.81 | 0.82 |
|  | Model 3 | 0.83 (0.74, 0.93) | **< 0.001** | 0.68 | 0.90 |
|  | Model 4 | 0.96 (0.92, 0.99) | **< 0.001** | 0.89 | 0.92 |
| MCI vs. NC | Model 1 | 0.89 (0.81, 0.97) | **< 0.001** | 0.88 | 0.84 |
|  | Model 2 | 0.79 (0.68, 0.90) | **< 0.001** | 0.65 | 0.81 |
|  | Model 3 | 0.69(0.56, 0.83) | **< 0.001** | 0.58 | 0.81 |
|  | Model 4 | 0.90 (0.82, 0.98) | **< 0.001** | 0.81 | 0.89 |

Model 1 adjusted for CBF and ATT of 7-delay in all ROIs; Model 2 adjusted for CBF of 7-delay in all ROIs; Model 3 adjusted for CBF of 1-delay in all ROIs; Model 4 adjusted for CBF, and ATT of 7-delay in all ROIs, sex, age, *APOE ε*4 carrier status and education years. Sensitivity and specificity are for cutpoints defined by Youden index (maximizing the sum of sensitivity and specificity). Abbreviations: NC, normal cognition; MCI, mild cognitive impairment; AD, Alzheimer’s disease; *APOE*, apolipoprotein E; ROI, region of interest; CBF, cerebral blood flow; ATT, arterial transit time; AUC, area under the curve; CI, confidence interval; ROC, receiver operating characteristic.

**Table S3** *P* values of ROC curves comparison in different models between diagnostic groups

|  | AD vs. NC | AD vs. MCI | MCI vs. NC |
| --- | --- | --- | --- |
| Model 1 vs. 2 | 0.66 | 0.15 | 0.06 |
| Model 1 vs. 3 | 0.47 | 0.21 | **0.008** |
| Model 1 vs. 4 | 0.41 | **0.04** | 0.67 |
| Model 2 vs. 3 | 0.43 | 0.97 | 0.25 |
| Model 2 vs. 4 | 0.53 | **0.008** | 0.06 |
| Model 3 vs. 4 | 0.14 | **0.007** | **0.005** |

Model 1 adjusted for CBF and ATT of 7-delay in all ROIs; Model 2 adjusted for CBF of 7-delay in all ROIs; Model 3 adjusted for CBF of 1-delay in all ROIs; Model 4 adjusted for CBF, and ATT of 7-delay in all ROIs, sex, age, *APOE ε*4 carrier status and education years. Abbreviations: NC, normal cognition; MCI, mild cognitive impairment; AD, Alzheimer’s disease; *APOE*, apolipoprotein E; ROI, region of interest; CBF, cerebral blood flow; ATT, arterial transit time; ROC, receiver operating characteristic.

**Table S4** Subgroup ROC analyses in CBF of 7-delay ASL for identifying different stages of AD

|  | AUC（95% CI） | | *p*-value^†^ |
| --- | --- | --- | --- |
|  | middle age | old age |  |
| AD vs. NC | 0.97 (0.84-1.00) ^*^ | 0.97 (0.85-1.00) ^*^ | > 0.05 |
| AD vs. MCI | 0.95 (0.82-0.99) ^*^ | 0.78 (0.62-0.89) ^*^ | > 0.05 |
| MCI vs. NC | 0.83 (0.68-0.92) ^*^ | 0.94 (0.74-0.99) ^*^ | > 0.05 |

Middle age group included 12 AD patients, 24 MCI patients and 19 NC subjects; old age group included 27 AD patients, 13 MCI patients and 7 NC subjects. Abbreviations: NC, normal cognition; MCI, mild cognitive impairment; AD, Alzheimer’s disease; ROI, region of interest; CBF, cerebral blood flow; AUC, area under the curve; CI, confidence interval; ROC, receiver operating characteristic.

^*^ *p* < 0.05.

^†^ *p* values of ROC curves comparison in age subgroups.
